# High-fat diet impairs cognitive function of zebrafish

**DOI:** 10.1038/s41598-019-53634-z

**Published:** 2019-11-19

**Authors:** Shinichi Meguro, Sayaka Hosoi, Takahiro Hasumura

**Affiliations:** 1 Biological Science Research, Kao Corporation, 2606 Akabane, Ichikai-machi, Haga-gun, Tochigi 321-3497 Japan; 2 Biological Science Research, Kao Corporation, 1334 Minato, Wakayama, Wakayama 640-8580 Japan

**Keywords:** Neurophysiology, Animal behaviour

## Abstract

An unhealthy diet with excessive fat intake has often been claimed to induce not only obesity but also cognitive dysfunction in mammals; however, it is not known whether this is the case in zebrafish. Here, we investigated the effect of excessive fat in the diet on cognitive function and on gene expression in the telencephalon of zebrafish. Cognitive function, as measured by active avoidance test, was impaired by feeding of a high-fat diet compared with a control diet. In RNA sequencing analysis of the telencephalon, 97 genes were identified with a fold change in expression greater than 2 and a *p*-value less than 0.05 between the two diets. In quantitative real-time PCR analysis of the telencephalon, genes related to neuronal activity, anti-oxidative stress, blood–brain barrier function and amyloid-β degradation were found to be downregulated, whereas genes related to apoptosis and amyloid-β production were found to be upregulated, in the high-fat diet group, which are changes known to occur in mammals fed a high-fat diet. Collectively, these results are similar to those found in mammals, suggesting that zebrafish can serve as a suitable animal model in research into cognitive impairment induced by excessive fat in the diet.

## Introduction

According to the World Health Organization, in December 2017 around 50 million people had dementia, and 10 million new cases emerge every year^1^. Dementia is a syndrome—usually of a chronic or progressive nature—in which there is deterioration in cognitive function^1^. Some research has shown a relationship between the development of cognitive impairment and life-style–related risk factors that are shared with other non-communicable diseases^1^. Obesity and unhealthy diet are two risk factors for dementia^1,2^. Excessive fat intake has often been claimed to induce obesity in humans and rodents^3,4^. Although numerous studies have been conducted to elucidate the effects of obesity and excessive dietary fat intake on cognitive function in rodents^5–7^, no such studies have been conducted in zebrafish (*Danio rerio*).

The organs and tissues of zebrafish are similar in terms of structure and function to those of humans. In addition, these fish are amenable to genetic manipulation, breed readily in captivity, and can be inexpensively maintained, making them a useful model organism for investigating many human pathological conditions^8^. Several reports suggest that zebrafish are a suitable model organism for examining the mechanisms underlying lipid metabolism leading to obesity. For example, overexpression of the endogenous melanocortin antagonist agouti-related protein or the serine/threonine protein kinase Akt1 results in the development of adiposity in zebrafish^9,10^. Overfeeding and a high-fat (HF) diet induce body fat accumulation in zebrafish through pathophysiological pathways common with those underlying mammalian obesity; furthermore, green tea extract and fish oil, which have well-known anti-obesity activities, can cancel out these effects. Thus, zebrafish are a useful model of human diet-induced obesity^11–14^, which led us to hypothesize that if a HF diet induces cognitive dysfunction in zebrafish, then zebrafish could also be a simple model for the screening of compounds with activity against dementia induced by unhealthy diet or obesity.

The zebrafish brain appears to be not only biochemically and functionally similar to that of mammals, but zebrafish also show complex cognition comparable to that seen in mammals^15–17^. There are a variety of learning test protocols for zebrafish based on pre-existing rodent protocols: e.g., active or passive avoidance test, Y-maze test, and T-maze test^15,17–23^. Aoki *et al*.^15^ found that active neural ensembles in the telencephalon are essential for the retrieval of long-term memories for learned behaviors in zebrafish. In addition, several reports examining learning protocols show that the *N*-methyl-d-aspartate receptor antagonist MK-801 and the cholinergic blocker scopolamine suppress cognitive function in zebrafish^19–23^. This means that the glutamatergic and cholinergic systems, which are well-known neurotransmitter systems involved in mammalian cognition, are also crucial in zebrafish. Furthermore, cognitive function decreases in aged zebrafish and increases in exercised zebrafish, which mirrors phenomena in humans^24–27^. However, the effect of diet on cognitive function in zebrafish is not well known.

Here, we investigated the effect of dietary fat on cognitive function in zebrafish. To understand the mechanism of this effect, we also conducted RNA sequencing and quantitative real-time PCR to examine the gene expression in the telencephalon of zebrafish.

## Results

### Effect of HF diet on body weight and body fat volume ratio in zebrafish

During the 11-week experimental period, no marked abnormalities or major differences were observed in feeding behavior between the diet groups (*n* = 16 in each group). Average energy intake of the HF diet group (HF group, 4.302 kcal/fish) was 11.2% greater than that of the control diet group (Cont group, 3.825 kcal/fish). There was no significant difference in body weight (mg) between the HF group (697.0 ± 37.0) and Cont group (777.6 ± 45.7). Body fat volume ratio, estimated as body fat volume (mm^3^) divided by body weight (mg), was slightly higher in the HF group (11.6 ± 1.9) than in the Cont group (7.5 ± 1.0), although this trend was not statistically significant (*p* = 0.084).

### Effect of HF diet on cognitive function in active avoidance test

**Figure 1:**
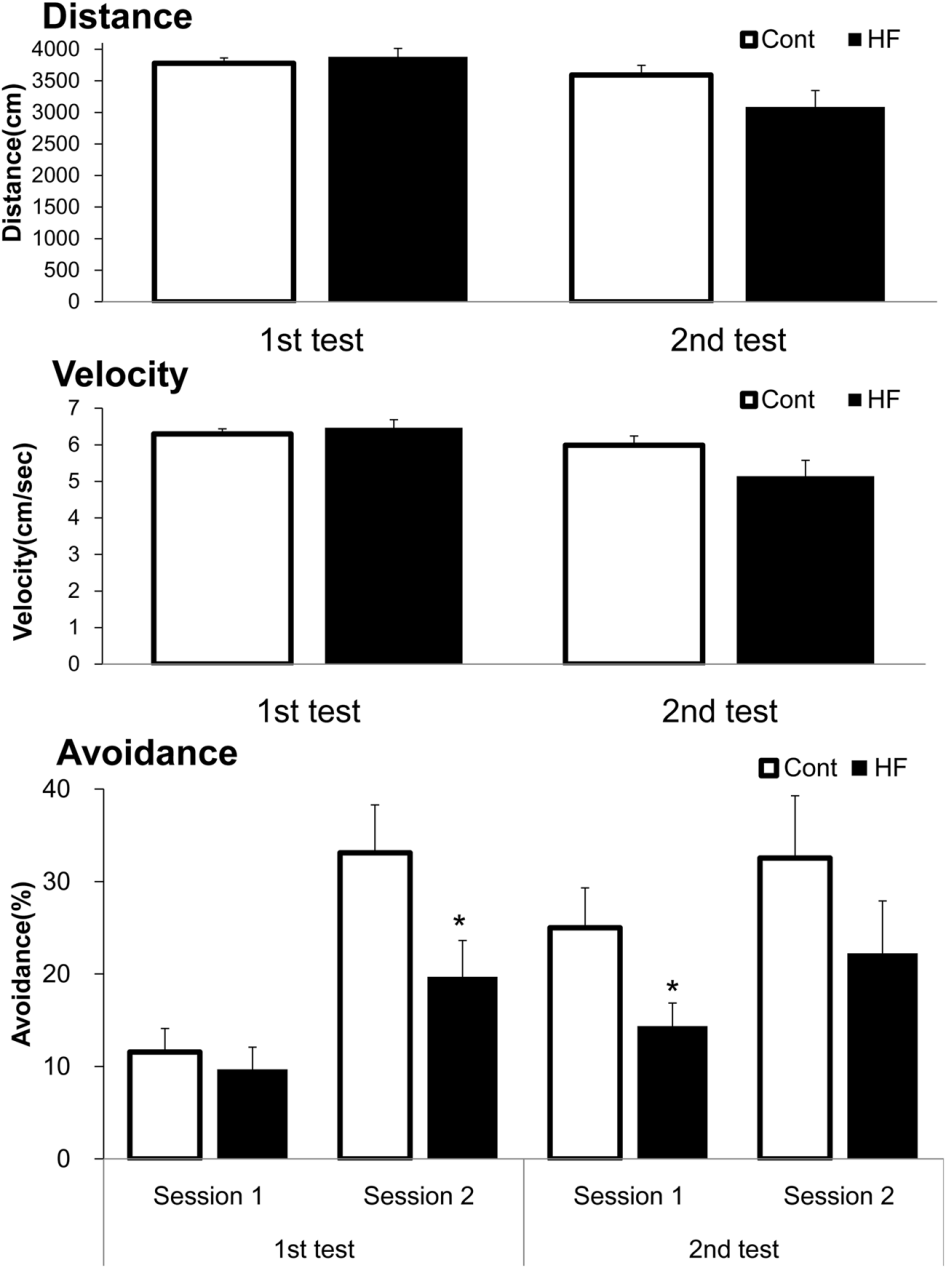
Effects of HF diet on spontaneous motor activity and avoidance in the active avoidance test. Zebrafish were fed Cont or HF diet for 8 weeks, and then subjected to an active avoidance test which performed once a week for two weeks (see Fig. 4A). In the latter half (10 min) of the first adaptation period, we measured spontaneous motor activity (distance [cm] and average velocity [cm/s]). In the session period, we measured avoidance (%) (avoidance of electric shock, see Fig. 4C). Results were analyzed by analysis of covariance (ANCOVA) using IBM SPSS Statistics Version 24 (IBM Corp., Armonk, NY, USA). Data are means ± standard error (*n = *16, *p < 0.05 vs. Cont group).

To confirm that the motor behaviors of the Cont and HF groups before the active avoidance tests, we recorded spontaneous motor activity (distance travelled and average velocity) in the latter half (10 min) of the first adaptation period. No significant differences between the Cont and HF groups were detected in distance (cm) or velocity (cm/sec) (Fig. 1). We then measured avoidance (%) to identify the effect of HF diet on cognitive function. Avoidance (%) in the HF group was significantly lower than that in the Cont group during session 2 in the first test and session 1 in the second test, and a similar trend was observed for session 1 in the first test and session 2 in the second test but was not statistically significant (Fig. 1). In addition, we measured the ratio of learner fish (i.e., fish that made eight avoidance responses in ten successive trials in session 1 of the first and second tests) to total fish. Learner fish ratio was significantly lower in the HF group than in the Cont group (Table 1).

**Table 1:** Effects of HF diet on Learner (%).

	Learner (%)^a^	
	First test	Second test
Cont	6.25	31.25
HF	12.50	12.50
*p*-value		0.011

^a^Fish that achieved the learning criterion by making eight avoidance responses in ten successive trials in session 1 of were considered “learners”.

### Effect of HF diet on mRNA expression levels in the telencephalon

**Figure 2:**
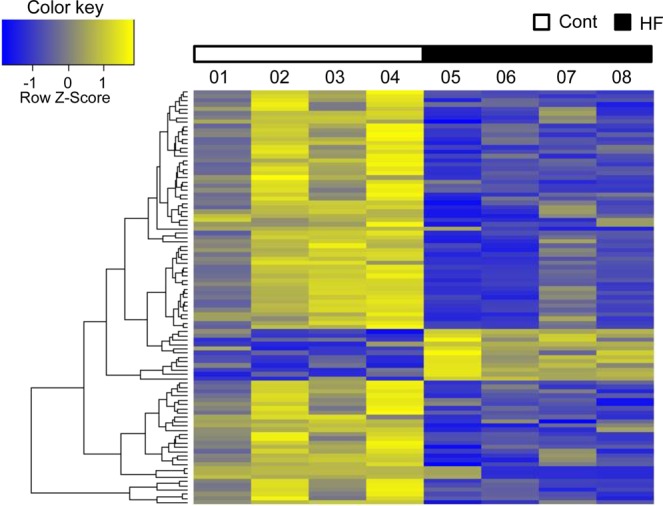
RNA sequencing analysis of the effects of HF diet on gene expression in the telencephalon in zebrafish. Total RNA was extracted from the telencephalon, and RNA sequencing of eight samples was conducted on the HiSeq. 2500 platform (*n* = 4 in each group). The figure shows a heat map of one-way hierarchical clustering of 97 genes satisfying the criteria of fold change >2 and *p* < 0.05 (HF vs. Cont group), constructed using Z-score as the normalized value (log2 based). The color key indicates the fold change between the individual gene transcript pairs. Relative expression of transcripts (rows) is displayed across individuals (columns) with yellow representing up-regulation and blue representing down-regulation in the HF group compared with the Cont group.

**Figure 3:**
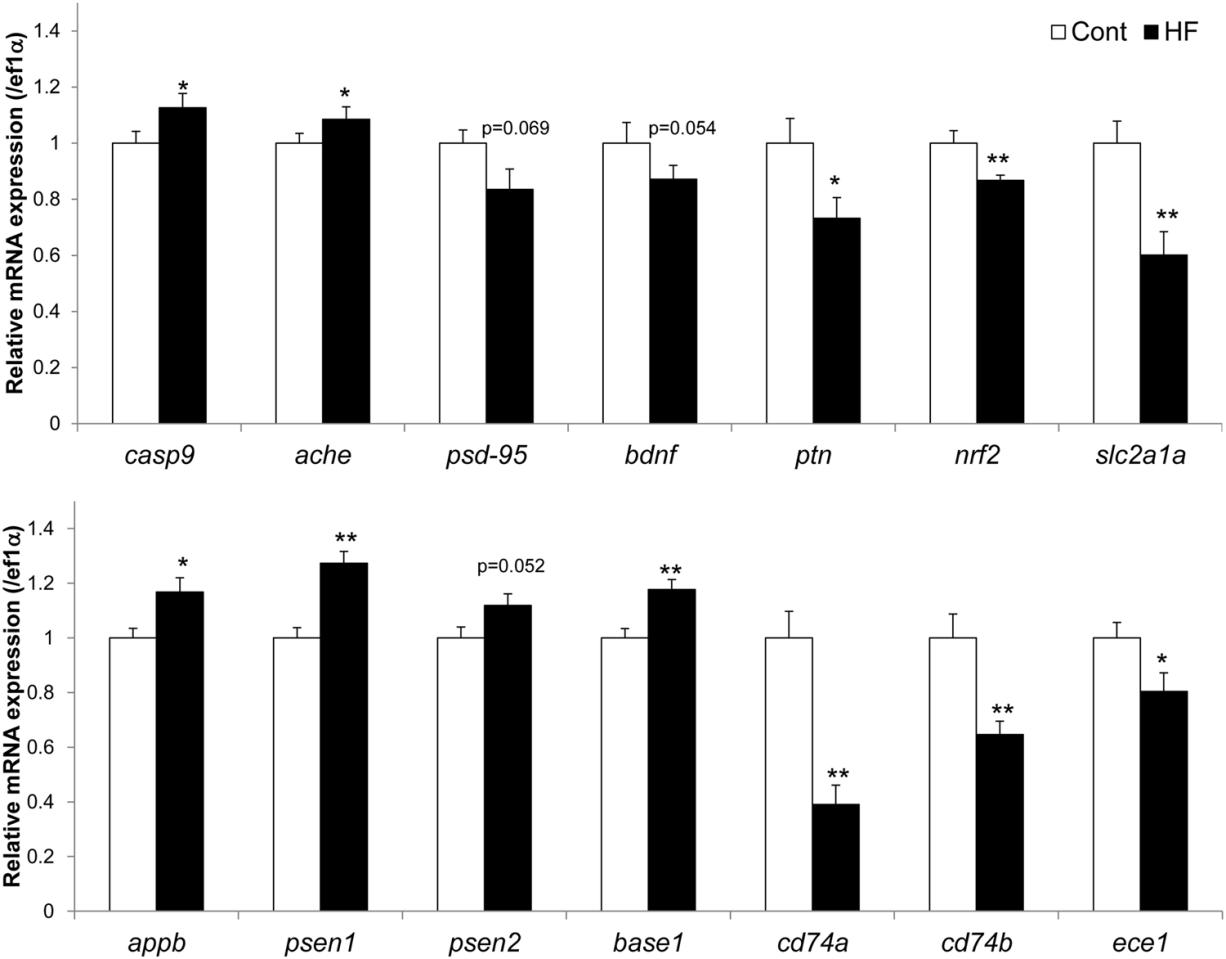
Effects of HF diet on relative mRNA expression levels in the telencephalon of zebrafish. The expression of each indicated mRNA was normalized against *ef1a* mRNA expression. Data are means ± standard error (*n* = 16, *p < 0.05, **p < 0.01).

To understand the molecular basis of the HF diet–induced cognitive impairment in zebrafish, we conducted RNA sequencing (*n* = 4 in each group) and quantitative real-time PCR analyses (*n* = 16 in each group) of the telencephalon. In the RNA sequencing analysis, using Z-score as the normalized value, 97 genes satisfied the criteria of fold change >2 and *p* < 0.05 for HF versus Cont groups by independent *t*-test (Fig. 2). Of these genes, 12 were up-regulated and 85 were down-regulated in the HF group compared with the Cont group. In the quantitative real-time PCR analysis, we measured genes known to be related to cognitive functions in mammals (Fig. 3). We found significantly higher levels of *casp9*, *ache*, *appb*, and *psen1* mRNAs and significantly lower levels of *ptn*, *nrf2*, *slc2a1a*, *cd74a*, *cd74b*, and *ece1* mRNAs in the HF group compared to the Cont group (Fig. 3); the level of *psen2* mRNA tended to be higher and those of *psd95* and *bdnf* mRNAs tended to be lower in the HF group than in the Cont group, but these differences were not statistically significant (*p* = 0.052, *p* = 0.069, and *p = *0.054, respectively).

## Discussion

Here we demonstrated that zebrafish display impaired cognitive function following consumption of a HF diet. Like mammals, zebrafish possess cognitive function, which is affected by neurotransmitters and so on^15–23^. However, there are no reports on the effect of the intake of fat as a macronutrient in the diet on cognitive function in zebrafish. Excessive fat intake, especially saturated fat, is recognized as a risk for cognitive impairment in humans^1,2,28–30^. Here, to produce the HF diet, we supplemented the basal diet with an excessive amount of lard, which is composed of a high percentage of saturated fatty acid^13,14^. Accordingly, HF diet–induced cognitive dysfunction in zebrafish might be similar to that in humans.

Because an unhealthy diet with excessive fat intake is a risk factor for induction of obesity, accumulated adipose tissue in the body has been assumed to be responsible for cognitive impairment in humans^1,2,28–31^. Zhou *et al*.^6^ reported that feeding a HF diet to male SAMP8 mice induced both adiposity and cognitive impairment, and McLean *et al*.^5^ showed that even one week of feeding of a HF diet to male C57Bl/6J mice increased their fat mass and impaired their episodic memory. Here, HF diet–fed male zebrafish tended to show a higher body fat volume ratio compared with Cont diet–fed ones, although this effect was not significant (*p* = 0.084). In our previous report, we showed that a HF diet, especially that containing lard (as used here), increases body fat volume in female zebrafish^13^. Collectively, these results suggest that HF diet–induced adiposity may accelerate cognitive impairment not only in mammals but also in zebrafish. We understand that further study is needed to clarify the relationship between adiposity and cognitive function in zebrafish.

Learner (%) in the first and second tests, which were conducted one week apart, was significantly suppressed in HF diet–fed zebrafish. Aoki *et al*.^15^ reported that learner fish, defined by criteria similar to those used here, show increased neural activity in the telencephalon compared with control fish. We therefore speculate that the telencephalon in HF diet–fed zebrafish might display suppressed neural activity, causing cognitive impairment. Further study is needed to understand the effect of HF diet on neural activity in the telencephalon of zebrafish.

No differences were observed in spontaneous motor activity, distance, and average velocity between the two test groups. For the active avoidance test, zebrafish have to be moved from the housing tank to a shuttle box, which is known to induce stress, an anxiety-like state, and spontaneous changes in motor activity^32,33^. Ideally, the condition of the fish should be comparable between groups to obtain precise results. Our findings regarding motor activity, distance, and average velocity suggest that the data obtained from the active avoidance test is reliable, and also that the HF diet likely had no effect on the psychiatric condition of the zebrafish.

The expression of 97 genes was altered in the telencephalon of HF diet–fed zebrafish compared with Cont diet–fed ones, according to the RNA sequencing analysis. We consider that these changes relate to cognitive changes in the HF diet–fed zebrafish. Recently, Huang *et al*.^34^ performed RNA sequencing analysis of the telencephalon in chronically stressed adult zebrafish and revealed the altered expression of 155 genes, some of which are known to be critical for memory. Although this chronic stress treatment did not include dietary stress, we consider that the HF diet might also produce memory-associated gene expression changes in the telencephalon of zebrafish similar to that in mammals^28–31,35–37^.

In the quantitative real-time PCR analysis, increased gene expression of *casp9* and *ache*, and decreased expression of *psd-95*, *bdnf*, *ptn*, *nrf2*, and *slc2a1a* were observed in the telencephalon of HF diet–fed zebrafish. These genes are well known regulators of neuronal, anti-oxidative, and blood–brain barrier functions for cognition, and the same changes are seen when cognitive impairment is induced in mammals^38–43^. For instance, a HF diet increases oxidative stress and decreases Nrf2 signaling with cognitive impairment in mice^41^. The mRNA levels of *slc2a1* (glucose transporter-1 gene) are decreased in the brain of HF diet–fed mice^42^ and in the subcutaneous adipose tissue of HF diet–fed humans^43^, and deletion of this gene accelerates cognitive impairment in mice^44^. However, the effects of these changes in gene expression are yet to be fully clarified in zebrafish brain. In addition, increased levels of *appb* and *psen1* mRNA, and decreased levels of *cd74a*, *cd74b*, and *ece1* mRNA in telencephalon were observed in HF diet–fed zebrafish. These changes in gene expression are considered to accelerate the production and suppress the degradation of amyloid-β protein in the brain of mammals^45,46^. Many reports show that amyloid-β protein is increased in the brain of HF diet–fed rodents with cognitive dysfunction^47–49^. However, the effects of increased levels of amyloid-β protein are yet to be fully clarified in zebrafish brain, with few studies having been conducted^50–52^. Further study is needed to clarify the functions of these genes in HF diet–fed zebrafish, although these findings do suggest that HF diet–induced cognitive impairment in zebrafish might be caused by changes in the expression of these genes.

Cognitive function was impaired in the HF group without an increase in body weight compared to the Cont group. Although the body fat volume ratio was slightly higher in the HF group than in the Cont group, this trend was not statistically significant (*p* = 0.084). We speculate that increased body fat volume might be more important for the induction of cognitive impairment in zebrafish than increased body weight. In rodents, HF diet–induced cognitive impairment is accompanied by both increased body weight and increased body fat^41,47^; however, we cannot directly extrapolate the rodent data to zebrafish. Further studies are needed to clarify the relationships between body weight and body fat volume and HF diet–induced cognitive impairment in zebrafish.

In the present study, we used only male zebrafish based on a study by Arslan-Ergul and Adams who reported differential gene expression between the brains of male and female zebrafish^53^. Male fish were chosen because in our laboratory we have found that body size within the same litter varies less than it does for female fish. However, it is possible that female fish will provide different results compared with those we obtained with male fish. Thus, a study in female fish is needed to resolve the sex differences of HF diet–induced impaired cognitive function in zebrafish.

In summary, zebrafish experienced impaired cognitive function following feeding of a HF diet, likely via down-regulation of genes related to neuronal activity, anti-oxidative stress, blood–brain barrier function, and amyloid-β degradation, and up-regulation of genes related to apoptosis and amyloid-β production. The functions of these genes and the effects of changes in their expression are well known in HF diet–fed mammals but not zebrafish. Thus, further studies are needed to clarify the functions of these genes and their changes in expression in HF diet–fed zebrafish. However, the present results are similar to those found in mammals, suggesting that zebrafish may serve as a suitable animal model for research into cognitive impairment induced by a diet rich in fat.

## Methods

### Animals

Zebrafish were purchased from a local pet supplier (Meito Suien Co., Ltd., Remix, Nagoya, Japan) and only the male offspring of the purchased fish were used. All the fish that were used were from the same litter and the average weight was around 400 mg at 3 months post-fertilization. All zebrafish were maintained on a 14-h light/10-h dark cycle at 28 °C, and water quality was kept within the guidelines in *The Zebrafish Book*^54^. All animal experiments were carried out in strict accordance with the regulations approved by the Animal Care and Experimentation Facility Committee of Kao Corporation (Tochigi, Japan) and with those outlined in *Guide for the Care and Use of Laboratory Animals*, 8th edition^55^.

### Diets

The Cont diet was freeze-dried standard zebrafish chow (Otohime B2; Marubeni Nissin Feed Co. Ltd., Tokyo, Japan). The HF diet was a mixture of 80% (w/w) Cont diet and 20% (w/w) lard (Oriental Yeast Co., Ltd., Tokyo, Japan), prepared by adding the Cont diet to the lard liquefied using a hot water bath and slow agitation. Table 2 shows the nutritional composition of the two diets.

**Table 2:** Nutritional composition of the Cont and HF diets.

	Cont	HF
**Ingredient (% w/w)**		
Lard	—	20
Basal diet^a^	100	80
**Nutrient (% w/w)**		
Carbohydrate	0	0
Protein	50	40
Fat	10	28
Ash and others	40	32
kcal/g diet^b^	3.790	4.912

^a^Basal diet was 100% Otohime B2.

^b^Calorific values were calculated using heat of combustion values for carbohydrate (4.20 cal/g), protein (5.70 cal/g), and fat (9.40 cal/g).

### Experimental design

Male adult zebrafish 3-months post-fertilization were anesthetized with 1.5 g/L tricaine (Sigma-Aldrich, St. Louis, MO, USA) solution containing 2 g/L NaHCO_3_ (Wako Pure Chemical Industries, Ltd, Osaka, Japan), weighed, and then allocated to two groups (*n* = 16 in each group) with similar body weights^56,57^. The fish were placed in 1.7-L tanks (8 fish per tank), and 40 mg of the experimental diet (Cont or HF) was added to each tank twice daily for 11 weeks. During feeding, the water inflow of the recirculation system of the zebrafish rack was paused for 1 h. Eight or nine weeks later, the fish were subjected to an active avoidance test. On the final day of the experiment, all fish were euthanized by prolonged immersion in tricaine, body weight was measured, and body fat volume was calculated with a three-dimensional micro-CT scanner (Rigaku Corporation, Tokyo, Japan), as previously described^13,14^. The telencephalon was then dissected from the rest of the brain and stored at −80 °C until RNA purification for determination of gene expression patterns.

### Measurement of food intake

Food intake monitoring was based on the protocol by Meguro *et al*.^13,14^. In brief, during the experimental period the amount of food consumed was scored visually 5 days each week on a 10-point scale from 0 (all food left) to 1.0 (no food left) by the same person who was blinded to the study design. The food intake of the eight fish in each tank was calculated as the amount fed multiplied by the food intake score (from 0 to 1.0). On the remaining 2 days of each week, the zebrafish were also fed, but food intake was not recorded. We therefore used the average score for the 3 days before and after the 2 days on which food intake was not scored. Energy intake was calculated from the food intake as the number of calories consumed per fish.

### Active avoidance test

**Figure 4:**
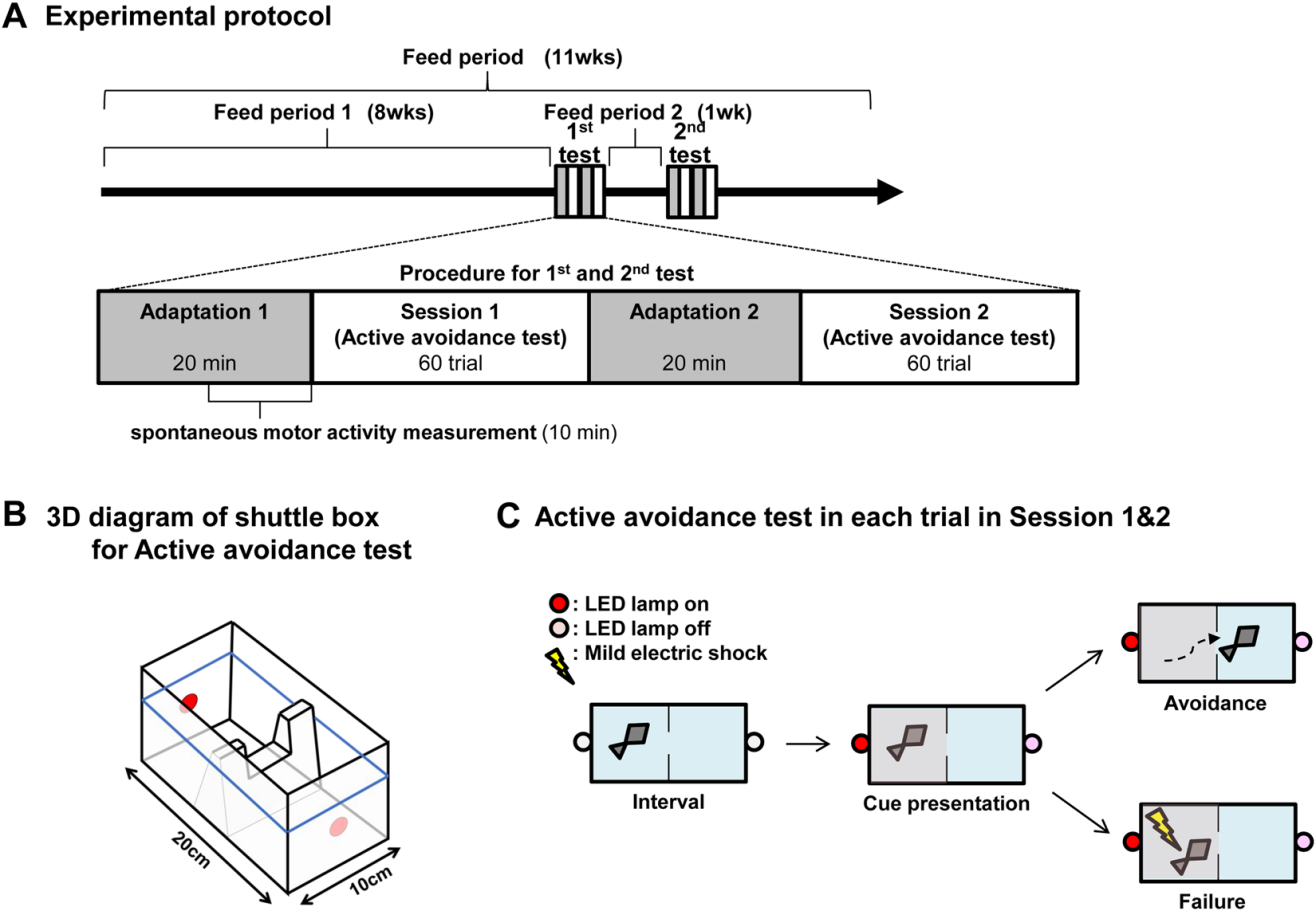
Paradigm of the active avoidance test in zebrafish. (**A**) During the 11-week feed periods, the active avoidance test was performed at 8 weeks (first test) and 9 weeks (second test). Each test comprised two adaptation periods (gray; 20 min each) and two session periods (white). In the latter half (10 min) of the first adaptation period, we measured spontaneous motor activity. (**B**) 3D diagram of the shuttle box (20 × 10 × 10 cm) for active avoidance test, filled with water (shown by blue line) from the recirculation system. The shuttle box was divided into two compartments of equal size by a hurdle. (**C**) The active avoidance test in each session contained 60 trials. Each trial comprised a 15-s interval prior to cue presentation and cue presentation in which the fish was given a cue in the form of a lit red LED lamp for 10 s followed by punishment by mild electric shock (2.5 V); the punishment was continued for 10 s, but was stopped when the zebrafish moved to the other compartment. “Avoidance” describes zebrafish that crossed hurdle without punishment. “Failure” describes zebrafish that not Avoidance.

An active avoidance test was performed once a week for two weeks after feed period 1, based on the protocol by Aoki *et al*.^15^. The test was performed during the daytime from 9:00 to 17:00, and the zebrafish were not fed until the test was completed. The test comprised two adaptation and two session periods. The adaptation comprised two 20-min periods, Adaptation 1 & 2. In the latter half (10 min) of the first adaptation period (Adaptation 1), we measured spontaneous motor activity (distance and average velocity). The session periods comprised two sessions, Session 1 & 2, with 60 trials in each session (Fig. 4A). Prior to the test, the zebrafish were gently transported using a fish net from the housing tank (24 × 6 × 14 cm, acrylic plastic) to a shuttle box (20 × 10 × 10 cm, acrylic plastic) filled with water (28 °C) from the recirculation system. The shuttle box was divided into two compartments of equal size by a hurdle and then placed in a soundproof chamber (Fig. 4B). In each traial, a single zebrafish was given a cue in the form of a lit red LED lamp for 10 s followed by punishment by mild electric shock (2.5 V). The punishment was continued for 10 s, but was stopped when the zebrafish moved to the other compartment. “Avoidance” describes zebrafish that crossed hurdle without punishment. “Failure” describes zebrafish that not Avoidance (Fig. 4C). Behavioral analysis was performed automatically using a LimeLight video tracking system (Actimetrics Inc., Wilmette, IL, USA). Zebrafish that achieved eight avoidance responses in ten successive trials in Session 1 of the first and second test were designated as a “Learner”.

### RNA extraction and sequencing

Total RNA was extracted from the frozen telencephalon of the zebrafish by using an RNeasy Lipid Tissue Mini Kit (Qiagen K.K., Tokyo, Japan). The high quality of the RNA (RIN, 8.4–9.6) for RNA sequencing, in eight samples (*n* = 4 in each group), was checked using an Agilent 4200 TapeStation system according to the manufacturer’s instructions (Agilent Technologies, Santa Clara, CA, USA). RNA sequencing transcriptome libraries were prepared with 100 ng of total RNA by using the TruSeq RNA Sample Preparation Kits v2 (Catalog# RS-122–2001, Illumina Inc., San Diego, CA, USA). Quality-controlled paired-end libraries were sequenced on the HiSeq. 2500 platform (Illumina Inc.). Read quality was assessed using FastQC (http://www.bioinformatics.babraham.ac.uk/projects/fastqc/). Trimming in RNA Sequencing data was performed with Trimmomatic version 0.32^58^. The alignment of the sequenced reads to the assembled transcriptome was performed with TopHat2 version 2.0.13, Bowtie version 2.2.2.3, and Cufflinks version 2.2.1^59–61^. FPKM (Fragments Per Kilobase of transcript per Million fragments mapped) was used as an indicator of gene expression level. In this study, only genes with a value of FPKM > 1 were considered to be expressed and were retained for expression comparisons. Therefore, from a total of 42,325 genes, only 22,697 were used for statistical analysis.

### Quantitative real-time PCR

RNA was transcribed into cDNA by using a High capacity RNA-to-cDNA Kit (Thermo Fisher Scientific Inc., Waltham, MA, USA). cDNA was analyzed by quantitative real-time PCR using the ViiA7 Real-Time PCR System (Thermo Fisher Scientific Inc., Waltham, MA, USA); analysis of cDNA samples employed TaqMan Fast Universal PCR Master Mix (Applied Biosystems). The TaqMan gene expression assays were as follows: *ef1a*, Dr03432748_m1; *casp9*, Dr03121787_m1; *ache*, Dr03093481_m1; *ptn*, Dr03071332_m1; *slc2a1a*, Dr03103605_m1; *nrf2*, Dr03131895_m1; *appb*, Dr03080308_m1; *psen1*, Dr03131346_m1; *psen2*, Dr03138298_m1; *bace1*, Dr03438714_m1; *cd74a*, Dr03150415_m1; *cd74b*, Dr03432831_m1; and *ece1*, Dr03130191_m1. The SYBR gene expression assays were as follows: *bdnf*, F: 5ʹ-ATAGTAACGAACAGGATGG-3ʹ, R: 5′-GCTCAGTCATGGGAGTCC-3′; *psd-95*, F: 5ʹ-GAGGATGTGATGCATGAGGATG-3ʹ, R: 5ʹ-TGTGTCCAGATGCGGAGAGT-3ʹ. Relative mRNA expression levels were determined by using the expression level of *ef1a* as the internal standard.

### Statistical analysis

All data are shown as means ± standard error. The avoidance number correlated with the crossing number in a shuttle box during the active avoidance test. Therefore, we used analysis of covariance (ANCOVA) for less influence by the crossing number, and calculated the avoidance score (%). Statistics were performed using analysis of variance followed by Fisher’s partial least-squares difference multiple comparison test. Differences between the proportions of learners (%) were examined by the chi square test. Statistical analyses were conducted using IBM SPSS Statistics Version 24 (IBM Corp., Armonk, NY, USA), and *p*-values less than 0.05 were considered statistically significant.

## Data Availability

The datasets generated and/or analyzed during the current study are available from the corresponding author on reasonable request.
